# Integrated Evaluation of Agronomic and Phytochemical Traits in Red Clover (*Trifolium pratense* L.) for Dual-Purpose Breeding

**DOI:** 10.3390/plants15121910

**Published:** 2026-06-20

**Authors:** Alexandru D. Costin, Andreea D. Ona, Zorița M. Diaconeasa, Floricuța Ranga, Anamaria Mălinaș, Ioana V. Berindean, Ionuț Racz, Mihai C. Popa, Leon Muntean

**Affiliations:** 1 Department of Genetics and Plant Breeding, Faculty of Agriculture, University of Agricultural Science and Veterinary Medicine of Cluj-Napoca, Calea Mănăștur Street No. 3–5, 400372 Cluj-Napoca, Romania; alexandru-daniel.costin@student.usamvcluj.ro (A.D.C.); ioana.berindean@usamvcluj.ro (I.V.B.); ionut.racz@usamvcluj.ro (I.R.); mihai.popa@usamvcluj.ro (M.C.P.); leon.muntean@usamvcluj.ro (L.M.); 2 Department of Food Science, Faculty of Food Science and Technology, University of Agricultural Science and Veterinary Medicine of Cluj-Napoca, Calea Mănăștur Street No. 3–5, 400372 Cluj-Napoca, Romania; zorita.sconta@usamvcluj.ro (Z.M.D.); floricutza_ro@yahoo.com (F.R.); 3 Department of Environmental Protection and Engineering, Faculty of Agriculture, University of Agricultural Sciences and Veterinary Medicine, Calea Mănăştur Street No. 3–5, 400372 Cluj-Napoca, Romania; anamaria.malinas@usamvcluj.ro; 4 Agricultural Research and Development Station Turda, 27 Agriculturii, 401100 Turda, Romania

**Keywords:** *Trifolium pratense*, forage productivity, polyphenols, antioxidant activity, multivariate analysis, flavonoids

## Abstract

Red clover (*Trifolium pratense* L.) is an important forage legume that is also a valuable source of bioactive compounds with potential health-promoting properties. This study evaluated the variability among diploid (2n) and tetraploid (4n) red clover cultivars in forage productivity, quality-related parameters, polyphenol and flavonoid content, and antioxidant activity, in order to identify promising ideotypes for dual-purpose breeding. A total of 90 cultivars were assessed under field conditions; green matter yield, dry matter yield, crude protein content, and protein yield were analyzed together with total polyphenols, total flavonoids, and antioxidant activity. Spearman correlation and principal component analysis (PCA) were used to relate the traits and identify cultivars with contrasting characteristics. Cultivar differentiation was pronounced within each ploidy group, whereas diploid and tetraploid cultivars overlapped substantially in the multivariate space, indicating that ploidy alone is not a reliable predictor of forage or medicinal value. At the group level, tetraploids tended toward higher biomass, protein-related traits, and total polyphenol concentration, while total flavonoids and antioxidant activity were broadly comparable between groups. Forage- and medicinal-related traits were only weakly correlated and thus behaved as largely independent selection targets—which is precisely why integrated multi-trait evaluation is required to identify cultivars combining both. Several cultivars did combine favorable agronomic and phytochemical characteristics, supporting within-group selection of red clover germplasm with dual forage and medicinal potential for sustainable agricultural systems.

## 1. Introduction

Red clover (*Trifolium pratense* L.) is an important perennial forage legume in temperate agriculture, primarily grown in short-term cultivated grasslands and grass–legume mixtures for cutting (hay, haylage, or silage) due to its high crude protein content and overall forage quality, and is widely considered a target crop for genetic improvement in forage systems [1,2]. Beyond its agronomic role, red clover is also recognized for medicinal and nutraceutical potential, largely attributed to its specialized metabolites, particularly isoflavones. Reviews and food/functional-ingredient studies consistently highlight major isoflavones such as biochanin A, formononetin, daidzein and genistein, which support much of the plant’s phytopharmacological and nutraceutical interest [3,4,5].

Breeding and cultivar evaluation commonly emphasize simultaneous improvement in biomass yield and forage quality, aiming for high CP [1,6].

Red clover is further recognized for phenolic compounds with potential health relevance. In phytochemical screening and functional-quality assessments, total polyphenols, total flavonoids, and antioxidant activity are widely used as integrative indicators of medicinal-related quality, while isoflavone-rich fractions have been discussed in relation to antioxidant properties and nutraceutical applications [4,5,7]. Importantly, red clover is encountered both as diploid (2n = 2x = 14) and tetraploid (4n) germplasm [1,8], and ploidy level is frequently examined as a source of variation in agronomic performance, forage quality, and phytochemical traits. Comparative studies indicate that tetraploids can differ in nutritive/structural composition, with reports of higher crude protein and lower lignin/unavailable carbohydrate fractions relative to diploids in specific cultivar comparisons [8], while other evaluations emphasize that biochemical patterns (including phenolics/isoflavones and antioxidant-related traits) can be environment- and material-dependent, motivating direct testing within defined cultivar panels and growing conditions [9,10]. Collectively, these observations support differentiated breeding goals and raise the possibility of dual-purpose selection schemes that combine forage performance with bioactive potential.

A practical limitation in cultivar recommendation and breeding is that productivity, forage quality (CP), and medicinal-related traits (e.g., polyphenols/flavonoids/antioxidant activity) are still often measured and interpreted in parallel streams, despite biological interdependence and shared trade-offs [3,6,10]. Multivariate approaches—particularly correlation analysis and principal component analysis (PCA)—are well-suited to integrate multi-trait data, identify coherent trait association patterns, and support ideotype-relevant grouping for purpose-driven parent selection [10,11].

In this context, the aim of our study was to evaluate the agronomic, forage, and medicinal-quality performance of 90 diploid and tetraploid red clover cultivars under the pedoclimatic conditions of the experimental site, in order to support breeding-oriented selection. Although the analytical methods employed are well-established in forage and phytochemical research, the novelty of the present study resides in the integrated assessment of a large red clover germplasm collection combining agronomic, forage-quality, phytochemical, and antioxidant-related traits within a unified breeding framework. Such comprehensive evaluations remain limited in red clover and are essential for identifying cultivars suitable for forage, medicinal, and dual-purpose breeding objectives.

To achieve this aim, the specific objectives were to: (i) quantify the relationships among productivity, forage quality, and medicinal-quality traits using correlation analysis; (ii) explore multivariate trait patterns and identify trait combinations relevant for cultivar differentiation and ideotype definition through principal component analysis (PCA); and (iii) identify high-performing cultivars with potential use as parental material for forage, medicinal, or dual-purpose breeding programs.

## 2. Results

### 2.1. Productivity and Protein-Related Forage Traits in Diploid and Tetraploid Cultivars

Substantial variation was observed among cultivars within both ploidy groups for green matter yield (GMY), dry matter yield (DMY), crude protein content (CP), and protein yield (PY), as shown in Table 1 for diploids (2n) and Table 2 for tetraploids (4n). Because DMY was estimated directly from GMY using a fixed conversion factor, the two biomass traits displayed closely parallel ranking patterns; therefore, GMY is presented primarily as a descriptive indicator of field productivity, whereas DMY was retained as the more biologically relevant dry-matter trait for integrative forage interpretation.

In the diploid group (Table 1), the highest biomass performance was recorded in Dimanche, Diadem, and Dizstende, all of which clearly exceeded the group mean for both GMY and DMY. However, the ranking for crude protein concentration differed from that for biomass accumulation, with AberClaret, AberChianti, and Otawa representing the leading cultivars for CP. This partial decoupling between yield and protein concentration is of direct breeding relevance, as it indicates that superior forage value cannot be inferred from biomass performance alone. To address this, protein yield (PY), calculated as the product of DMY and CP, was used as an integrative criterion that captures both forage productivity and protein concentration. Under this criterion, Diadem emerged as the strongest overall diploid entry, followed by AberChianti and Tășnad, while Dimanche, Otawa, and Discovery also ranked among the most promising materials. These results show that elite diploid performance may arise either from very high biomass production, from elevated protein concentration, or from a favorable balance between the two components.

A similarly differentiated pattern was observed in the tetraploid group (Table 2). Bivoj, Sigord, Vesna, Dolina, and Beskyd formed the upper tail for biomass production, with Bivoj showing the highest DMY. For CP concentration, Bivoj again ranked first, followed by Vesna and Tornado, indicating that some tetraploid cultivars combined strong biomass accumulation with improved protein content. This pattern became even clearer when evaluated through PY, where Bivoj was the leading cultivar, followed by Vesna and Sigord, while Beskyd and Dolina also remained above the tetraploid mean. Thus, in tetraploids as in diploids, the most agronomically valuable entries were not defined solely by high yield or high protein concentration in isolation, but rather by the extent to which both components were successfully combined.

Overall, the mean comparisons and significance coding support a clear breeding interpretation. In both ploidy groups, cultivar differentiation was pronounced for all four traits, but the highest-biomass cultivars were not always those with the highest CP. Consequently, PY represents the most informative integrative criterion for identifying superior forage candidates, because it reflects the combined contribution of dry matter productivity and protein concentration. From a breeding perspective, cultivars with high PY should be prioritized as forage-oriented candidates, whereas cultivars with exceptionally high CP but less competitive PY remain valuable as donor parents for improving forage quality in future crossing schemes. Within this framework, Diadem and AberChianti were the most promising diploid entries, whereas Bivoj, Vesna, and Sigord represented the strongest tetraploid candidates.

### 2.2. Medicinal Quality Traits and Bioactive Productivity in Diploid and Tetraploid Cultivars

Marked cultivar-dependent variation was observed within both ploidy groups for all medicinal-related traits, including total polyphenols, total flavonoids, antioxidant activity, and their corresponding per-plant outputs (Table 3 for diploids and Table 4 for tetraploids). In the present study, medicinal potential was interpreted at two complementary levels. First, concentration-based traits—total polyphenols, total flavonoids, and antioxidant activity expressed per unit mass—were used to characterize biochemical quality per se. Second, the corresponding per-plant values were used as integrative indicators of bioactive productivity, analogous in concept to protein yield in forage evaluation, because they combine biochemical concentration with head weight per plant (HWP). This distinction is biologically and breeding-relevant, as cultivars with high concentration are not necessarily those with the highest total delivery of bioactive compounds at the plant level.

At the group-mean level, tetraploid cultivars tended to show higher total polyphenol concentration than diploids, whereas total flavonoid concentration was broadly comparable between ploidy groups and antioxidant activity showed no consistent ploidy-related advantage. This pattern suggests that ploidy may influence specific branches of secondary metabolism, but that medicinal value remains strongly genotype-dependent within each ploidy group rather than being determined by ploidy alone.

In diploids, medicinal selection clearly separated concentration-focused donor genotypes from high-output candidates (Table 3). For concentration-based donor potential, Radviliai was the strongest entry for both total polyphenols and total flavonoids, while Dizstende and Diplo also ranked among the leading cultivars for polyphenol concentration. Antioxidant activity per gram was highest in Livada Ralu, Raunis, and Granta, with several additional cultivars showing similarly elevated values. However, when medicinal performance was interpreted through per-plant bioactive productivity, the ranking changed substantially. Diplo, Callisto (C1), and AberClaret were the leading cultivars for total polyphenols per plant, indicating that high medicinal output may arise either from elevated concentration or from a favorable balance between concentration and reproductive biomass. A similar integrative pattern was observed for antioxidant output per plant, where Verdi was the strongest diploid entry, followed by David Liv, GKT Junior, and Raunis. For flavonoid productivity per plant, AberClaret emerged as the most valuable diploid genotype, with Callisto (C2) and Radviliai also ranking among the top-performing entries. Collectively, these results indicate that diploid medicinal ideotypes can be differentiated into elite donor cultivars for biochemical richness and elite candidates for total bioactive delivery.

A similarly differentiated pattern was observed in tetraploids (Table 4). For concentration-based medicinal quality, Ilte, Poljanka, and Bivoj formed the leading group for total polyphenol concentration, whereas Linus, Lasang, and Dolina displayed the highest antioxidant activity per gram. Total flavonoid concentration was led by Legato, followed by Sadunai and Beskyd. However, as in diploids, the most informative breeding interpretation emerged from the integrated per-plant traits. Bivoj was the strongest tetraploid genotype for total polyphenols per plant, followed by Sadunai, Vesna, and Tornado, confirming that superior medicinal output depends not only on concentration, but also on the capacity to sustain greater head biomass. For antioxidant activity per plant, Dolina was the leading cultivar, with Magura and Lasang forming the next most relevant group. For flavonoid output per plant, Sadunai ranked first, followed by Tornado and Bivoj. These results show that tetraploid medicinal value is likewise multidimensional, with some cultivars acting primarily as concentration donors and others as superior high-output candidates.

Overall, the mean comparisons and significance coding support a clear breeding interpretation. Concentration-based traits remain essential for identifying donor parents with intrinsically enriched phytochemical profiles, whereas per-plant bioactive productivity provides the most informative integrative criterion for selecting medicinally valuable cultivars under field conditions, because it captures the combined effect of biochemical concentration and head biomass. From this perspective, Radviliai, Diplo, Callisto (C1), AberClaret, and Verdi were among the most relevant diploid medicinal candidates, while Bivoj, Sadunai, Dolina, Tornado, and Vesna represented the strongest tetraploid entries, depending on whether the breeding objective prioritizes concentration, output, or a balanced combination of both.

### 2.3. Trait Associations

**Figure 1:**
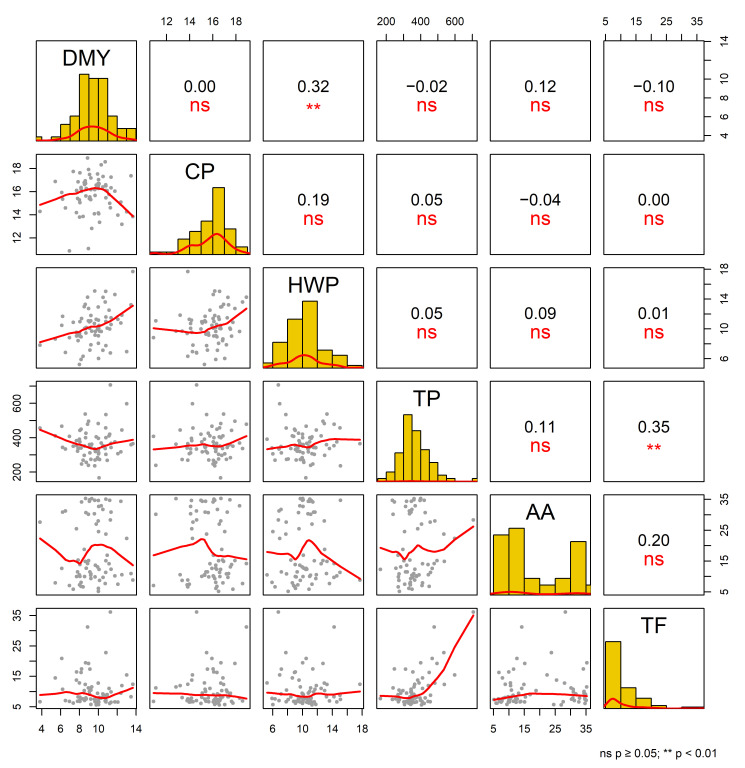
Spearman correlation matrix among forage, capitulum, and medicinal traits in diploid red clover cultivars (2n). Abbreviations: DMY, dry matter yield; CP, crude protein; HWP, head weight per plant; TP, total polyphenols; AA, antioxidant activity; TF, total flavonoids.

In diploid cultivars (2n), Spearman correlations based on cultivar means revealed generally weak associations between forage-related traits (dry matter yield, DMY; crude protein, CP) and capitulum/medicinal traits (head weight per plant, HWP; total polyphenols, TP; antioxidant activity, AA; total flavonoids, TF) (Figure 1). The only significant inter-group relationship was a modest positive correlation between DMY and HWP (ρ = 0.32, *p* < 0.01), indicating that higher dry biomass tended to coincide with heavier capitula in diploid materials. Within the medicinal trait set, TP was positively correlated with TF (ρ = 0.35, *p* < 0.01), whereas AA showed no significant association with either TP or TF. CP was not significantly correlated with HWP, TP, AA, or TF, suggesting that protein concentration varied largely independently of bioactivity-related traits in diploids. Overall, these results support the view that forage performance and medicinal quality can be treated as partially independent selection targets in diploid cultivars.

**Figure 2:**
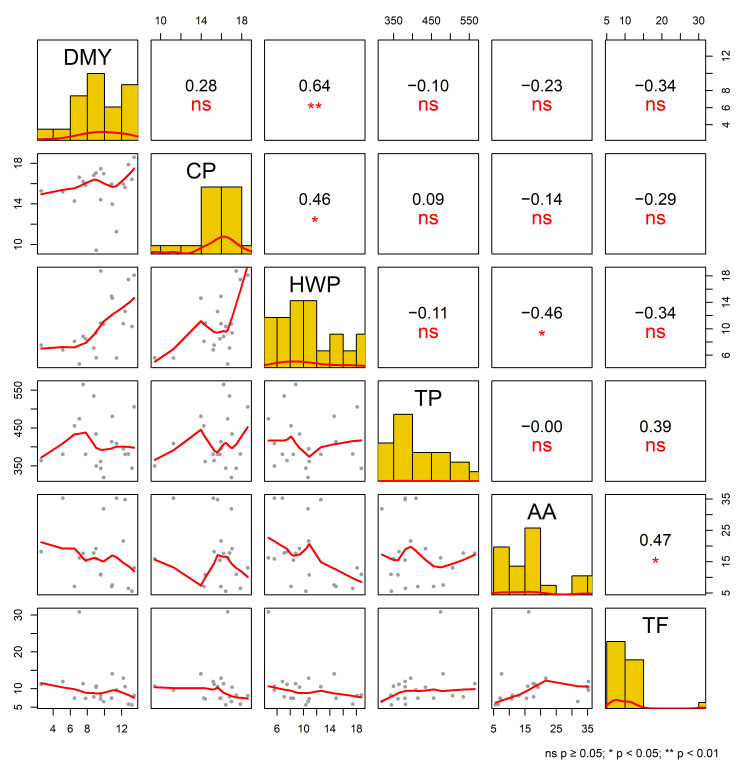
Spearman correlation matrix among forage, capitulum, and medicinal traits in tetraploid red clover cultivars (4n). Abbreviations: DMY, dry matter yield; CP, crude protein; HWP, head weight per plant; TP, total polyphenols; AA, antioxidant activity; TF, total flavonoids.

In tetraploid cultivars (4n), Spearman correlations based on cultivar means also indicated generally weak associations between forage-related traits and medicinal traits, although several significant relationships were centered on head weight per plant (HWP) (Figure 2). Dry matter yield (DMY) was positively correlated with HWP (ρ = 0.64, *p* < 0.01), indicating that higher dry biomass production tended to coincide with heavier capitula in tetraploid materials. Crude protein (CP) was also positively associated with HWP (ρ = 0.46, *p* < 0.05), suggesting that cultivars with heavier capitula tended to display higher protein concentration. Within the medicinal trait set, antioxidant activity (AA) was positively correlated with total flavonoids (TF) (ρ = 0.47, *p* < 0.05), whereas total polyphenols (TP) showed no significant association with AA. HWP was negatively correlated with AA (ρ = −0.46, *p* < 0.05), indicating an inverse relationship between capitulum biomass and antioxidant capacity. Overall, these results suggest that, in tetraploids, dual-purpose selection should jointly consider biomass-related traits and antioxidant potential, as gains in capitulum weight and forage performance may not necessarily be accompanied by increased antioxidant activity.

### 2.4. Principal Component Analysis (PCA)

**Figure 3:**
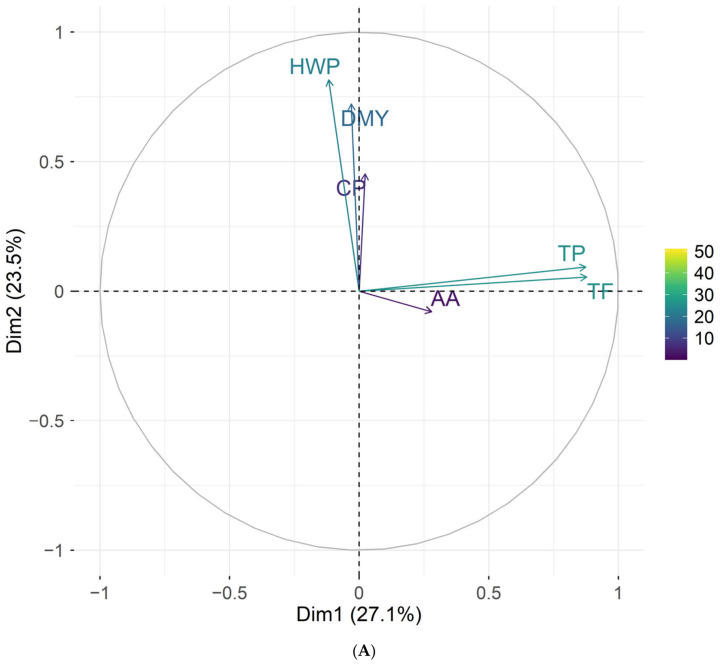

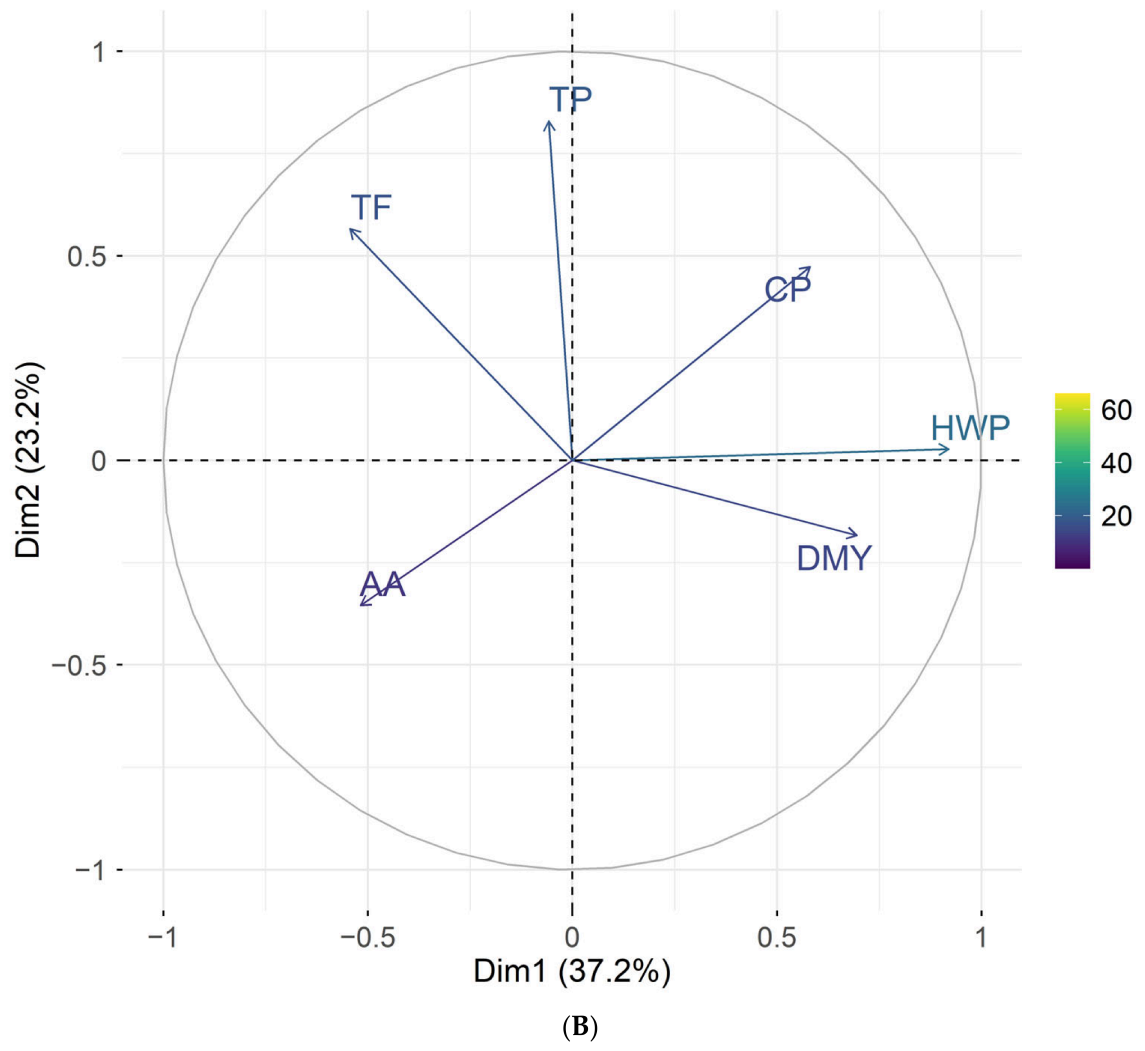
PCA variable contribution for: (**A**) diploid red clover cultivars (2n); (**B**) tetraploid red clover cultivars (4n). Abbreviations: DMY, dry matter yield; CP, crude protein; HWP, head weight per plant; TP, total polyphenols; AA, antioxidant activity; TF, total flavonoids; Dim1 and Dim2 represent the first and second principal components, explaining 27.1% and 23.5% of the total variance, respectively.

Principal component analysis revealed distinct patterns of trait covariation in the diploid germplasm. In diploids (Figure 3A), Dim1 (27.1%) was driven mainly by the phytochemical block, with total polyphenols (TP) and total flavonoids (TF) showing long, nearly collinear vectors aligned with the positive side of the axis, indicating a tight positive association between these two variables. In contrast, Dim2 (23.5%) was influenced primarily by head weight per plant (HWP), while dry matter yield (DMY) and crude protein (CP) were oriented in the same positive direction but with shorter vectors, suggesting a weaker yet concordant contribution to this secondary axis. Antioxidant activity (AA) was positioned on the positive side of Dim1 but slightly negative on Dim2, indicating that AA did not behave as a direct indicator of total phenolic accumulation. Overall, the diploid ordination supports a two-domain structure in which phenolic composition and productivity-related traits are separated in the PC1–PC2 plane, while antioxidant activity represents an additional functional dimension.

In tetraploid cultivars (Figure 3B), the PCA configuration indicated a clear reorganization of trait covariation relative to diploids. Dim1 (37.2%) was associated primarily with productivity-related traits, with head weight per plant (HWP) showing the strongest positive loading and dry matter yield (DMY) also contributing positively to this axis, although with a negative association on Dim2. Crude protein (CP) was positioned in the positive quadrant, indicating partial alignment with the productivity-related gradient and a simultaneous contribution to the second dimension. In contrast to the diploid pattern, the medicinal traits did not converge on a single common axis in tetraploids. Total polyphenols (TP) loaded mainly on the positive side of Dim2 with minimal contribution to Dim1, whereas total flavonoids (TF) were oriented toward negative Dim1 and positive Dim2, indicating a distinct covariance structure relative to the yield-related traits. Antioxidant activity (AA) occupied the negative quadrant on both axes, confirming that it represented a separate functional component and was not redundant with either total phenolics or productivity variables in the PC1–PC2 space. Overall, the tetraploid ordination suggests that productivity, phenolic composition, and antioxidant activity were only partially coupled, and therefore ideotype-based selection in 4n germplasm should treat these components as partially independent breeding targets.

**Figure 4:**
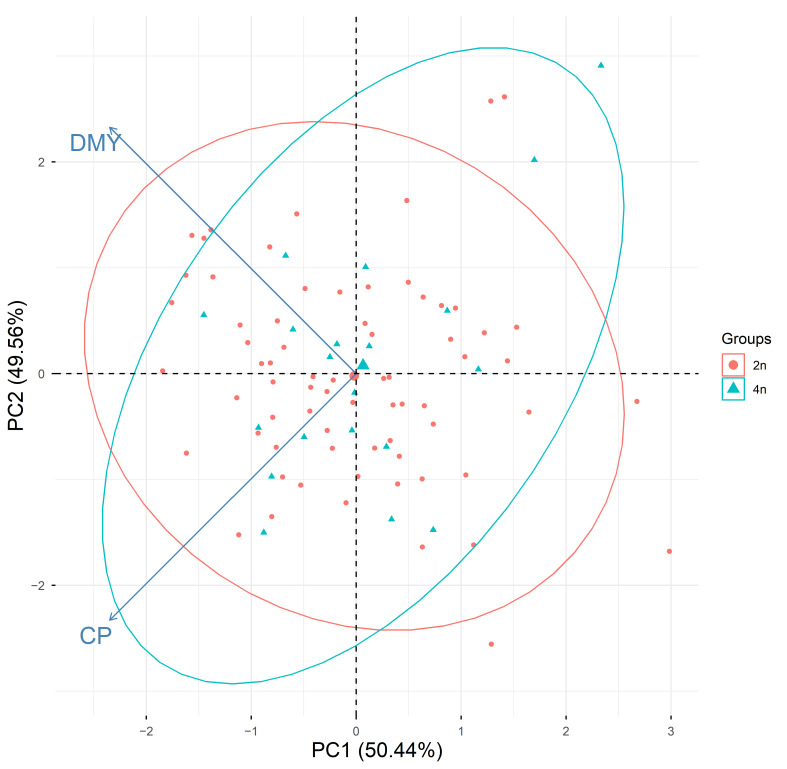
Combined PCA biplot for diploid (2n) and tetraploid (4n) red clover cultivars based on forage-related traits. Abbreviations: DMY, dry matter yield; CP, crude protein; PC1 and PC2 represent the first and second principal components, explaining 50.44% and 49.56% of the total variance, respectively.

The combined PCA biplot for forage-related traits (Figure 4) showed no clear ploidy-based separation between diploid and tetraploid cultivars. The first two principal components together accounted for 100% of the variance in this two-variable dataset (PC1 = 50.44%, PC2 = 49.56%). Dry matter yield (DMY) and crude protein (CP) showed a common negative orientation on PC1, whereas PC2 separated cultivars toward relatively higher DMY or relatively higher CP, indicating a contrasting distribution along the yield–protein gradient in the ordination space. The confidence ellipses of the two ploidy groups overlapped substantially, suggesting that ploidy status alone did not generate discrete forage-performance clusters in this material. Instead, cultivar differentiation was driven mainly by relative positioning along the DMY and CP axes, supporting selection within, rather than between, ploidy groups for forage-related ideotypes.

**Figure 5:**
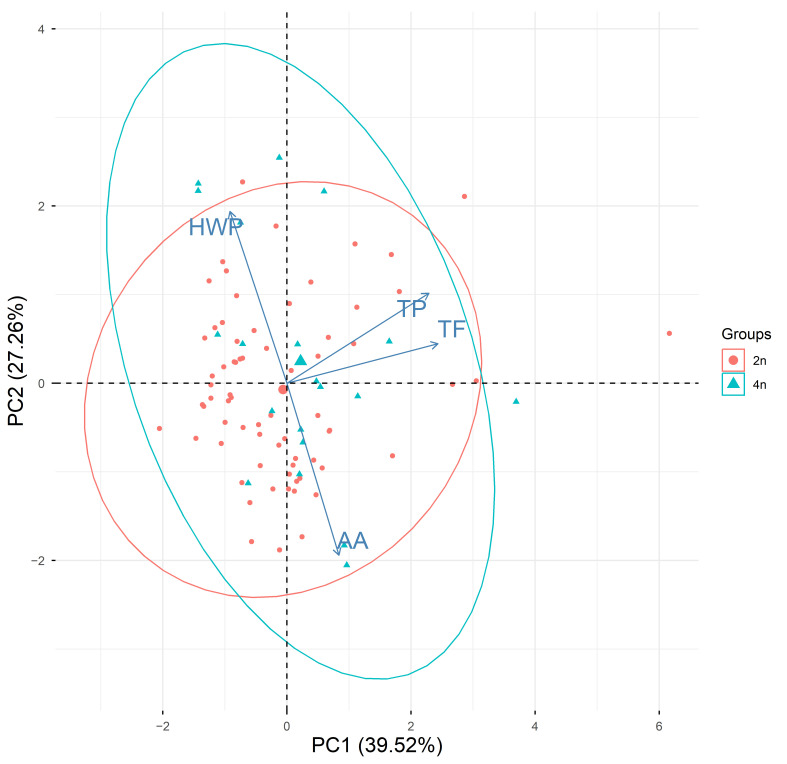
Combined PCA biplot for diploid (2n) and tetraploid (4n) red clover cultivars based on medicinally relevant traits. Abbreviations: DMY, dry matter yield; CP, crude protein; PC1 and PC2 represent the first and second principal components, explaining 50.44% and 49.56% of the total variance, respectively.

The combined PCA biplot for medicinally relevant traits (Figure 5) showed substantial overlap between diploid and tetraploid cultivars, indicating that ploidy status alone did not produce a clear multivariate separation in the ordination space. The first two principal components explained a substantial proportion of the total variance (PC1 = 39.52%, PC2 = 27.26%). Total polyphenols (TP) and total flavonoids (TF) were oriented in a broadly similar direction along the positive side of PC1, supporting the presence of a shared phenolic-composition gradient. In contrast, antioxidant activity (AA) projected toward the negative side of PC2, indicating that it represented a partially distinct functional dimension rather than a direct surrogate of total phenolic accumulation. Head weight per plant (HWP) was oriented separately from the main phenolic vectors, suggesting that reproductive biomass and medicinal composition were not expressed as a single integrated gradient at cultivar level. The broad overlap of the confidence ellipses further indicates that cultivar differentiation was driven more by trait syndromes than by ploidy per se, supporting ideotype-based selection within both cytological groups.

### 2.5. Breeding-Oriented Cultivar Recommendations

Based on integrated trait analysis, cultivars were classified according to their suitability for specific breeding and utilization objectives. Distinct groups of genotypes were identified, including high-biomass cultivars suitable for forage production, high-protein genotypes for nutritional improvement, and high-polyphenol cultivars with enhanced medicinal potential.

Importantly, a subset of cultivars exhibited a balanced combination of agronomic and phytochemical traits, making them particularly suitable for dual-purpose breeding strategies targeting both forage yield and functional compound production. These classifications provide practical selection guidance for breeding programs and germplasm utilization. The summary presented in Table 5 provides a practical framework for selecting red clover cultivars according to specific breeding objectives, including forage production, nutritional quality, and phytochemical value, as well as dual-purpose utilization.

## 3. Discussion

The present study confirms that red clover should not be evaluated solely as a conventional forage legume, because its breeding value emerges from the combined expression of productivity, forage quality, and medicinally relevant traits.

A particular strength of the present work is the integrated evaluation of a large germplasm collection comprising 90 diploid and tetraploid cultivars assessed simultaneously for agronomic, forage-quality, phytochemical, and antioxidant traits. Such comprehensive multi-trait evaluations remain relatively scarce in red clover and provide a valuable basis for ideotype-oriented breeding and cultivar selection.

This integrated perspective is consistent with the broader view that temperate forage legumes should be improved through multi-trait breeding strategies that better match target use and production context [1,3,4,5,6,12]. In the present material, this was particularly evident because cultivar differentiation was shaped more by trait syndromes than by ploidy alone, indicating that breeding decisions should focus primarily on genotype-specific combinations of desirable traits rather than on a simple diploid–tetraploid dichotomy.

A first important outcome of the study is that most practically useful variation occurred within ploidy groups rather than between them. From a breeding perspective, this finding is particularly valuable because it demonstrates that ploidy level alone cannot be used as a surrogate criterion for cultivar selection and highlights the importance of direct multi-trait phenotypic evaluation. This agrees with previous work showing that red clover exhibits substantial diversity for agronomic, nutritive, and phytochemical traits at cultivar or population level, and that ploidy effects, although informative, are not sufficiently deterministic to replace direct phenotypic evaluation [6,8,9,10,13,14]. The substantial overlap of diploid and tetraploid cultivars in the combined PCA biplots supports this interpretation directly. Thus, ploidy remains a biologically relevant source of variation, but it does not constitute a reliable stand-alone predictor of forage or medicinal ideotype value in a breeding-oriented germplasm collection such as the one studied here.

The forage-related results further show that crude protein concentration and protein yield should not be treated as interchangeable criteria. In forage legumes, breeding value depends not only on the concentration of nutritive constituents, but also on the amount of harvested biomass in which these constituents are expressed [6,13,15,16]. In the present material, some cultivars appeared superior mainly because of elevated CP concentration, whereas others gained importance through the combination of acceptable CP with stronger DMY and therefore higher PY. This distinction is essential from a breeding standpoint. A genotype may be a useful donor for improved protein concentration without being optimal for harvested protein output, whereas superiority for PY better captures the practical value of a cultivar under biomass-oriented forage use. The present results therefore support the use of PY as the most informative integrative criterion for forage-oriented selection, particularly when the breeding target is a productive material with favorable nutritive return under field conditions.

The medicinally relevant results reveal a similarly important level of diversity. Red clover is well-known as a source of phenolics and isoflavones with nutritional, antioxidant, and phytopharmacological relevance [3,4,5,8]. However, the present data also indicate that total polyphenols, total flavonoids, and antioxidant activity should not be interpreted as fully redundant descriptors of medicinal value. This interpretation is fully consistent with the broader phytochemical literature, in which the total phenolic content and antioxidant activity may covary, but not necessarily in a fixed or linear way, because antioxidant performance depends on compositional structure, dominant compound classes, plant part, and extraction context [4,5,7,14,17]. Antioxidant capacity is inherently method-dependent: assays such as DPPH, FRAP, ORAC, and CUPRAC differ in reaction mechanism (single-electron-transfer versus hydrogen-atom-transfer), pH, and sensitivity to different antioxidant classes. The ABTS results reported here should therefore be interpreted as one robust comparative measure of antioxidant potential rather than an exhaustive estimate; complementary multi-assay characterization would further refine the antioxidant ranking of cultivars in future work. Accordingly, medicinal screening based only on total polyphenols would risk oversimplifying the biological meaning of cultivar differences.

An especially relevant outcome of the present study is the distinction between concentration-based medicinal quality and biomass-linked bioactive productivity. A cultivar may show high levels of TP, TF, or AA on a tissue basis without producing the highest total amount of bioactive compounds at the plant level, whereas another genotype may achieve greater overall delivery through stronger head development combined with moderate-to-high biochemical concentration. Conceptually, this mirrors the distinction between crude protein concentration and protein yield in forage evaluation. Such a distinction is highly relevant for dual-purpose breeding, because breeding objectives may differ according to end use: one scheme may prioritize phytochemical richness per unit biomass, whereas another may target the total output of bioactive raw material. In this context, the negative association observed in tetraploids between HWP and AA suggests that larger reproductive biomass does not automatically imply greater antioxidant potential, and that direct balancing among targets may be necessary in medicinal or dual-purpose selection schemes.

Detailed compound-level phenolic profiling of these cultivars, obtained by HPLC-DAD-ESI-MS, has been previously reported [9]; the present study focused on the spectrophotometric medicinal-quality indicators—total polyphenols, total flavonoids, and antioxidant activity—across the full cultivar panel. This approach is consistent with recent studies employing spectrophotometric assays for the comparative evaluation of antioxidant-rich plant materials and agricultural by-products [18].

The correlation analysis supports a biologically meaningful contrast between diploids and tetraploids. In diploids, the generally weak cross-domain associations suggest a more modular trait structure, which is favorable for breeding because it implies a degree of independence between forage-related and medicinally relevant traits. In tetraploids, in contrast, several significant relationships were centered on head weight per plant, linking this trait positively with DMY and CP but negatively with AA. This pattern suggests a more constrained covariance structure in 4n germplasm and implies that dual-purpose selection may be somewhat more demanding in tetraploids. Comparative phytochemical studies also indicate that ploidy can influence red clover composition, including total phenolics and isoflavones, although such effects remain trait-dependent and should be interpreted in relation to genotype background and plant organ rather than as universal rules [8,9,10,14].

The PCA results further strengthen this interpretation. In diploids, TP and TF defined a clearer phytochemical axis, whereas productivity-related traits occupied a different region of the ordination space and AA behaved as an additional functional dimension. In tetraploids, the covariance pattern was reorganized, with stronger productivity loading on the main axis and weaker convergence among medicinal traits. This indicates that the underlying architecture of trait covariation differs between cytological groups. At the same time, the combined PCA showed substantial overlap between 2n and 4n germplasm, which argues against rigid ploidy-based classification of breeding potential. Similar conclusions have been reported in recent diversity and stability studies, where cultivar- or population-level variability often outweighed simple categorical grouping by cytological status [13,14,19].

Taken together, the results support the definition of three practical breeding directions in red clover: a forage-oriented ideotype with high DMY and favorable CP/PY; a medicinal-oriented ideotype with elevated TP, TF, and/or AA, interpreted either on a concentration basis or through total bioactive output; and a dual-purpose ideotype combining acceptable agronomic performance with enhanced medicinal potential. The present data suggest that this third category is biologically realistic, but will likely be identified through targeted multi-trait selection rather than through single-trait ranking or ploidy-based expectation alone. In this sense, the study supports the use of ideotype-oriented selection frameworks for red clover breeding, consistent with broader recommendations in forage-legume improvement [12].

Several limitations should nevertheless be considered. The medicinal characterization was based mainly on global spectrophotometric indicators, which are highly useful for comparative screening but less specific than full compound-resolved profiling. In addition, the integrative per-plant medicinal traits depend partly on head weight per plant and therefore reflect bioactive productivity at the plant level rather than direct plot-scale harvest output. Finally, DMY was treated here as an estimated trait derived from GMY, which is acceptable for comparative breeding analysis but should be borne in mind when interpreting the exact independence among productivity-related variables. Even with these limitations, the study clearly demonstrates that red clover germplasm contains exploitable diversity for the joint improvement of forage value and medicinal potential, and that cultivar-specific trait combinations provide a more informative basis for parent choice than ploidy alone.

Overall, the findings indicate that red clover breeding can benefit from a genuinely integrated framework in which productivity, protein-related forage value, and bioactive potential are evaluated jointly. Rather than treating diploids and tetraploids as inherently separate breeding categories, the present results support selection within both groups for ideotype-compatible cultivar syndromes relevant to forage, medicinal, or dual-purpose use [1,3,4,5,6,12,13,14,16,17,19].

## 4. Materials and Methods

### 4.1. Plant Material and Experimental Design

The germplasm collection comprised 90 red clover (*Trifolium pratense* L.) cultivars originating from a wide range of breeding programs across Europe, North America, and South America. The collection included 70 diploid and 20 tetraploid cultivars. Cultivars were obtained from various breeding institutions and germplasm sources, including Aberystwyth University (United Kingdom), DLF Seeds (Denmark/Czech Republic), Agri Obtentions (France), Agroscope (Switzerland), Graminor AS (Norway), SCDA Livada (Agricultural Research and Development Station Livada, Romania), and other national breeding and research organizations. The cultivars were selected to represent broad geographical diversity and different ploidy levels, enabling the evaluation of adaptation, agronomic performance, forage quality, and phytochemical traits under the environmental conditions of Cluj-Napoca, Romania. Detailed information regarding cultivar origin, country of development, ploidy level, and source institution is provided in Supplementary Table S1. The collection was treated as a fixed (non-random) set for comparative, breeding-oriented screening. Accordingly, the experiment is best interpreted as a replicated comparative germplasm screening trial with non-randomized but permuted cultivar layouts, rather than as a classical randomized complete block design (RCBD). This structure was considered appropriate because the study targeted a fixed cultivar collection for breeding-oriented comparison rather than statistical inference to a random genotype population, and cultivar order was deliberately changed among replications to reduce systematic positional bias associated with a single repeated field arrangement.

The field experiment was conducted in the Agro-Botanical Garden located on the campus of the University of Agricultural Sciences and Veterinary Medicine (UASVM) in Cluj-Napoca, Romania (46°46′ N, 23°36′ E, 340 m a.s.l.). The site is characterized by a temperate continental climate. Agronomic management followed standard regional practices for red clover throughout the experimental period.

The trial was established by sowing on 11 May 2021. The experiment included three field replications. Within each field replication (plot), trait measurements were based on five independent subsamples (individual plants/measurement units), and the reported value for each replication corresponds to the arithmetic mean of these five measurements. In each replication, each cultivar was grown on a 1 m^2^ plot with 12.5 cm inter-row spacing (eight rows per plot). Plots were separated by 30 cm alleys, and replications were separated by 50 cm alleys. To reduce systematic field-position effects associated with a single repeated ordering pattern, cultivar order differed among replications using three predefined layout schemes: (i) alphabetical order, (ii) grouping by country of origin, and (iii) grouping by geographical zone (i.e., the cultivar sequence was permuted among replications rather than repeated identically).

Trait assessments were performed in 2022, corresponding to the second year of stand life, when red clover typically reaches its main productive phase after establishment. Measurements were conducted according to standard phenological stages. Meteorological data (daily precipitation and air temperature) were obtained from the UASVM on-campus meteorological station. Annual precipitation totals and mean annual air temperature were computed for 2022. Hydrothermal anomalies were calculated relative to the 30-year station reference period (1990–2020). In 2022, the total annual precipitation was 533.4 mm (−173.6 mm vs. the 1990–2020 mean of 707 mm), and the mean annual air temperature was 10.6 °C (+0.8 °C vs. 9.8 °C).

### 4.2. Trait Measurements

Forage-quality and medicinal-quality analyses were performed on dried and ground plant material collected during the second year of stand development (2022), at the early flowering stage (approximately 50% flowering), from each biological replicate. Green matter yield (GMY) was recorded at approximately 50% flowering by weighing the harvested herbage and expressing yield on an area basis (kg m^−2^), with values readily convertible to t ha^−1^. Dry matter yield (DMY) was treated as an estimated trait derived from GMY using a fixed fresh-to-dry conversion of 4:1 (≈25% dry matter), consistent with the commonly reported dry matter content of freshly cut red clover herbage at a comparable developmental stage [6,8]. Because DMY represented a deterministic linear transformation of GMY rather than an independently measured variable, the two traits were not treated as independent inputs in multivariate analyses; accordingly, GMY was retained for a descriptive presentation of productivity, whereas DMY was used as the biomass-yield variable in correlation analysis, PCA, and other trait-integration procedures. Head weight per plant (HWP) was determined at peak flowering as the mass of collected flower heads per plant (g plant^−1^). All productivity measurements were performed at the same phenological stage to ensure comparability across cultivars.

Forage-quality analyses were performed on dried and ground plant material collected at the optimal harvest stage from each biological replicate. Crude protein content was determined for the full cultivar collection using the Kjeldahl method described by Dhont and Vanden Berghe [20].

Medicinal-quality indicators were assessed independently for each biological replicate. Total polyphenols (TP) were quantified spectrophotometrically using the Folin–Ciocalteu method [21] and expressed as mg gallic acid equivalents per 100 g dry matter (mg GAE 100 g^−1^). Total flavonoids (TF) were determined using the aluminum chloride colorimetric method [22] and expressed as mg quercetin equivalents per 100 g dry matter (mg QE 100 g^−1^). Antioxidant activity (AA) was evaluated using the ABTS radical scavenging assay [23] and expressed as µM Trolox equivalents per gram dry matter (µM Trolox g^−1^).

The ABTS•^+^ radical-cation assay was selected because its stable, water- and organic-solvent-compatible radical responds to both hydrophilic and lipophilic antioxidants across a broad pH range, making it well-suited to the structurally diverse phenolics, flavonoids, and isoflavonoids of methanolic red clover extracts; the method is also rapid, reproducible, and offers a wide linear range, and it ensured consistency with the spectrophotometric protocols adapted from [24].

All spectrophotometric determinations were carried out in the authors’ laboratory on samples from the 2022 field trial, each processed independently for every biological replicate. Total flavonoids and antioxidant activity are reported here for the first time; the total-polyphenol values derive from the 2022 screening of this panel, also reported at group level in [9], and are presented here per cultivar.

The total polyphenols per plant (TPP), total flavonoids per plant (TFP), and antioxidant activity per plant (AAP) were calculated by integrating the concentration-based measurements with HWP, thereby providing an estimate of the total amount of bioactive compounds and antioxidant capacity produced per plant.

This unit scaling was applied to enable the integration of phytochemical traits with biomass yield, allowing for the assessment of total bioactive compound production per plant, which is more relevant for breeding-oriented evaluation than concentration values alone.

### 4.3. Statistical Analysis

The analyzed variables were summarized as mean ± standard deviation (SD). Distributional assumptions were assessed using Q–Q plots and normality tests (Kolmogorov–Smirnov for larger samples and Shapiro–Wilk for smaller samples).

Based on data distribution, comparisons between individual cultivars and the overall collection mean were conducted using one-way ANOVA or Welch’s ANOVA for normally distributed data, and the Kruskal–Wallis test for non-Gaussian data. In this framework, each cultivar mean (based on biological replicates) was compared against the overall (collection-wide) mean for the same trait within the same year. When the overall test indicated statistically significant differences, post hoc comparisons were performed using Dunnett’s test or Dunn’s test, as appropriate. Statistical significance was set at *p* < 0.05.

To examine inter-trait relationships by ploidy, Spearman correlations were computed and visualized as scatterplot matrix (pairs plot). Only traits representing non-redundant, collection-scale information were retained for correlation analysis and multivariate ordination. Because DMY was calculated directly from GMY using a fixed conversion factor, these two biomass variables were not analyzed simultaneously as independent traits; DMY was retained as the biomass-yield variable for trait integration, whereas GMY was used only for a descriptive presentation of the productivity results.

Principal component analysis (PCA) was then applied as a complementary multivariate tool after checking data suitability with Bartlett’s test of sphericity and the Kaiser–Meyer–Olkin (KMO) index. Prior to PCA, variables were standardized (mean-centered and scaled to unit variance). Because the study aimed to compare covariance structure both within ploidy groups and within functional trait blocks, four PCA configurations were analyzed using the R package ‘factoextra’ [25]: (i) a diploid-group PCA based on DMY, CP, HWP, TP, TF, and AA; (ii) a tetraploid-group PCA based on the same trait set; (iii) a combined PCA across both ploidy groups for forage-related traits (DMY and CP); and (iv) a combined PCA across both ploidy groups for medicinally relevant traits (HWP, TP, TF, and AA).

All statistical analyses were performed using R software (version 4.5.2) [26].

## 5. Conclusions

This study demonstrated substantial variability among the evaluated diploid and tetraploid red clover cultivars in forage productivity, quality-related parameters, and the accumulation of bioactive compounds. Importantly, this variability was expressed mainly within each ploidy group rather than between diploids and tetraploids, and the multivariate analyses showed substantial overlap between the two cytological groups. Ploidy therefore does not constitute a reliable stand-alone predictor of forage or medicinal value, and selection is best performed within, rather than between, ploidy groups. At the group level, tetraploid cultivars tended toward higher biomass production, crude protein content, protein yield, and total polyphenol concentration, supporting their use in forage-oriented breeding, whereas total flavonoids and antioxidant activity were broadly comparable between ploidy groups.

The integration of agronomic and phytochemical traits through correlation analysis and principal component analysis showed that forage-related and medicinal-related traits were only weakly correlated and therefore behaved as largely independent selection targets. Rather than limiting the value of the approach, this partial independence is precisely what makes integrated multi-trait evaluation necessary, because cultivars combining both forage and medicinal value cannot be identified by ranking on any single trait. Several cultivars did combine acceptable forage characteristics with favorable bioactive profiles and may therefore represent valuable genetic resources for dual-purpose breeding.

On this basis, three practical breeding directions can be defined. For forage-oriented breeding, the most promising candidates were Diadem and AberChianti among diploids and Bivoj, Vesna, and Sigord among tetraploids; for medicinal-oriented breeding, Radviliai, Diplo, and AberClaret (diploids) and Bivoj, Sadunai, and Tornado (tetraploids) stood out for phenolic richness or bioactive output; and as dual-purpose candidates, Bivoj and Vesna (tetraploids), together with Callisto (C1) and Dimanche (diploids), combined acceptable agronomic performance with favorable bioactive profiles. Overall, the findings support the use of integrated multi-trait evaluation and within-group selection in red clover breeding and contribute to the development of cultivars adapted to sustainable agricultural systems, with enhanced value both as forage crops and as sources of biologically active compounds.

## Figures and Tables

**Table 1:** Productivity and forage-quality traits of diploid cultivars.

Cultivar	Green Matter Yield GMY (t/ha)	Dry Matter Yield DMY (t/ha)	Crude Protein CP (% DM)	Protein Yield PY (t/ha)
Mean ($\overset{-}{X}$)–Control (Ct.)	37.77 ± 7.32	9.44 ± 1.83	15.77 ± 1.56	1.49 ± 0.32
AberChianti	42.90 ± 0.99	10.73 ± 0.25	18.57 ± 0.28 ***	1.99 ± 0.02 *
AberClaret	35.44 ± 7.01	8.86 ± 1.75	18.90 ± 0.34 ***	1.67 ± 0.30
Aita Mare	31.20 ± 1.13	7.80 ± 0.28	15.41 ± 0.30	1.20 ± 0.07
Arimaiciai	43.20 ± 0.57	10.80 ± 0.14	15.47 ± 0.15	1.67 ± 0.04
Britta	38.74 ± 10.10	9.69 ± 2.52	16.26 ± 0.44	1.57 ± 0.37
Callisto (C1)	43.16 ± 4.58	10.79 ± 1.15	16.59 ± 0.19 *	1.79 ± 0.17
Callisto (C2)	35.88 ± 3.73	8.97 ± 0.93	17.91 ± 0.34 ***	1.61 ± 0.14
Corvus	24.48 ± 3.68	6.12 ± 0.92 *	15.93 ± 0.19	0.98 ± 0.16 *
David Liv	32.90 ± 8.80	8.23 ± 2.20	16.03 ± 0.56	1.32 ± 0.40
Diadem	53.92 ± 6.90 *	13.48 ± 1.73 ***	17.37 ± 0.48 ***	2.34 ± 0.36 ***
Dimanche	54.68 ± 5.09 *	13.67 ± 1.27 ***	13.85 ± 0.20 ***	1.89 ± 0.15
Diplo	34.42 ± 5.06	8.61 ± 1.27	16.94 ± 0.49 ***	1.46 ± 0.26
Diplomat	44.58 ± 2.12	11.15 ± 0.53	15.76 ± 0.31	1.76 ± 0.05
Dipper	38.60 ± 3.11	9.65 ± 0.78	15.66 ± 0.44	1.51 ± 0.08
Discovery	47.00 ± 1.41	11.75 ± 0.35	16.04 ± 0.12	1.88 ± 0.07
Dizstende	52.08 ± 2.72 *	13.02 ± 0.68 **	14.25 ± 0.36 ***	1.86 ± 0.14
Essi	40.28 ± 2.38	10.07 ± 0.59	16.68 ± 0.18 **	1.68 ± 0.08
Flora	35.76 ± 9.28	8.94 ± 2.32	11.09 ± 0.21 ***	0.99 ± 0.28 *
Gandalf	15.22 ± 8.46	3.81 ± 2.11 ***	14.29 ± 0.40 ***	0.54 ± 0.31 ***
GKT Junior	40.00 ± 4.53	10.00 ± 1.13	14.16 ± 0.45 ***	1.42 ± 0.21
Gloria Mestnaia	40.20 ± 1.41	10.05 ± 0.35	14.09 ± 0.29 ***	1.42 ± 0.08
Granta	37.20 ± 2.83	9.30 ± 0.71	12.83 ± 0.22 ***	1.19 ± 0.07
Justin	27.74 ± 7.16	6.94 ± 1.79	10.89 ± 0.08 ***	0.75 ± 0.19 ***
Kindia	50.00 ± 2.83 *	12.50 ± 0.71 *	13.18 ± 0.45 ***	1.65 ± 0.04
Kornicevskij	39.70 ± 0.42	9.93 ± 0.11	17.52 ± 0.18 ***	1.74 ± 0.01
Kuhn	48.32 ± 0.68	12.08 ± 0.17	13.98 ± 0.53 ***	1.69 ± 0.09
L.E. 116	31.40 ± 4.24	7.85 ± 1.06	16.85 ± 0.43 ***	1.32 ± 0.15
Leliceni	36.84 ± 8.37	9.21 ± 2.09	16.17 ± 0.43	1.49 ± 0.30
Lemmon	40.06 ± 6.87	10.02 ± 1.72	16.51 ± 0.26 *	1.65 ± 0.26
Livada Ralu	34.60 ± 8.77	8.65 ± 2.19	16.70 ± 0.36 ***	1.44 ± 0.34
Livada Sara	39.96 ± 1.19	9.99 ± 0.30	16.70 ± 0.30 **	1.67 ± 0.02
Lucrum	24.82 ± 3.37	6.21 ± 0.84 *	15.35 ± 0.39	0.95 ± 0.15 **
Manuela	40.10 ± 1.27	10.03 ± 0.32	17.03 ± 0.04 ***	1.71 ± 0.06
Marcom	34.90 ± 0.42	8.73 ± 0.11	16.26 ± 0.34	1.42 ± 0.05
Marga Liv	44.86 ± 8.40	11.22 ± 2.10	14.99 ± 0.18 *	1.68 ± 0.30
Marieta	32.00 ± 6.79	8.00 ± 1.70	15.28 ± 0.36	1.22 ± 0.29
Marino	32.06 ± 7.83	8.02 ± 1.96	13.79 ± 0.25 ***	1.11 ± 0.25
Mercury	33.54 ± 8.00	8.39 ± 2.00	13.85 ± 0.17 ***	1.16 ± 0.26
Merian	26.04 ± 7.30	6.51 ± 1.82	14.59 ± 0.21 ***	0.95 ± 0.28 **
Merviot (C1)	32.64 ± 7.01	8.16 ± 1.75	16.25 ± 0.31	1.33 ± 0.31
Merviot (C2)	41.94 ± 7.44	10.49 ± 1.86	16.40 ± 0.47	1.72 ± 0.35
Mestecăniș	43.80 ± 1.41	10.95 ± 0.35	16.06 ± 0.25	1.76 ± 0.08
Metis	32.18 ± 6.53	8.05 ± 1.63	15.06 ± 0.44	1.21 ± 0.21
Mistral	49.60 ± 7.92	12.40 ± 1.98 *	15.10 ± 0.48	1.87 ± 0.24
Montcalme	21.72 ± 3.79	5.43 ± 0.95 ***	16.88 ± 0.35 ***	0.92 ± 0.18 **
Nemaro	36.40 ± 5.09	9.10 ± 1.27	15.55 ± 0.54	1.41 ± 0.15
Niderheicher	38.32 ± 10.07	9.58 ± 2.52	14.09 ± 0.23 ***	1.35 ± 0.38
Noe	36.80 ± 11.88	9.20 ± 2.97	15.31 ± 0.35	1.41 ± 0.49
Oden Walder	40.06 ± 8.57	10.02 ± 2.14	16.27 ± 0.75	1.63 ± 0.28
Otawa	41.20 ± 2.83	10.30 ± 0.71	18.32 ± 0.42 ***	1.89 ± 0.17
Palna	36.20 ± 4.24	9.05 ± 1.06	15.39 ± 0.28	1.39 ± 0.14
Pavo	34.72 ± 5.77	8.68 ± 1.44	15.62 ± 0.36	1.36 ± 0.19
Podjavorina	32.66 ± 8.46	8.17 ± 2.11	16.36 ± 0.30	1.34 ± 0.32
Radviliai	45.14 ± 10.10	11.29 ± 2.52	14.60 ± 0.62 ***	1.65 ± 0.44
Raunis	43.24 ± 0.06	10.81 ± 0.01	16.10 ± 0.63	1.74 ± 0.07
Reichersberger	43.20 ± 3.96	10.80 ± 0.99	16.50 ± 0.51 *	1.78 ± 0.22
Rotrif	29.36 ± 11.54	7.34 ± 2.88	17.74 ± 0.39 ***	1.30 ± 0.49
Sapporo	30.68 ± 7.18	7.67 ± 1.80	14.18 ± 0.48 ***	1.09 ± 0.22
Segur	34.00 ± 9.05	8.50 ± 2.26	16.77 ± 0.08 ***	1.43 ± 0.39
Sepia	34.16 ± 5.32	8.54 ± 1.33	16.62 ± 0.59 *	1.42 ± 0.27
Sinope	36.28 ± 0.74	9.07 ± 0.18	17.48 ± 0.54 ***	1.59 ± 0.02
Slatina	45.40 ± 12.73	11.35 ± 3.18	16.33 ± 0.14	1.85 ± 0.53
Suez	30.80 ± 4.53	7.70 ± 1.13	16.09 ± 0.14	1.24 ± 0.19
Tabor	36.84 ± 1.19	9.21 ± 0.30	17.18 ± 0.23 ***	1.58 ± 0.07
Tășnad	45.40 ± 5.37	11.35 ± 1.34	17.25 ± 0.39 ***	1.96 ± 0.19 *
Tinu Liv	39.10 ± 3.25	9.78 ± 0.81	16.56 ± 0.21 *	1.62 ± 0.16
Verdi	38.44 ± 8.54	9.61 ± 2.14	16.17 ± 0.28	1.55 ± 0.32
Violeta	40.52 ± 5.15	10.13 ± 1.29	13.36 ± 0.57 ***	1.35 ± 0.11
Violetta R.v.P.	42.00 ± 10.18	10.50 ± 2.55	17.31 ± 0.37 ***	1.82 ± 0.40
Vyciai	32.20 ± 5.94	8.05 ± 1.48	17.63 ± 0.47 ***	1.42 ± 0.30
*p*-value	<0.001	<0.001	<0.001	<0.001

C1 & C2 (Callisto and Merviot) are the same cultivar received from different centers. Values presented as mean ± SD; */**/***—significance from post hoc tests according to 0.05/0.01/0.001 significance threshold.

**Table 2:** Productivity and forage-quality traits of tetraploid cultivars.

Cultivar	Green Matter Yield GMY (t/ha)	Dry Matter Yield DMY (t/ha)	Crude Protein CP (%DM)	Protein Yield PY (t/ha)
Mean ($\bar{X}$)–Control (Ct.)	37.93 ± 11.36	9.48 ± 2.84	15.56 ± 2.16	1.50 ± 0.54
Amos	36.00 ± 6.22	9.00 ± 1.56	17.04 ± 0.21 ***	1.58 ± 0.25
Beskyd	48.80 ± 3.96	12.20 ± 0.99	15.96 ± 0.54	1.98 ± 0.09
Bivoj	53.86 ± 10.55	13.47 ± 2.64 *	18.59 ± 0.44 ***	2.50 ± 0.55
Dolina	49.68 ± 1.24	12.42 ± 0.31	15.61 ± 0.55	1.92 ± 0.02
Ilte	30.04 ± 0.06	7.51 ± 0.01	16.21 ± 0.26	1.22 ± 0.02
Lars	10.40 ± 2.83 *	2.60 ± 0.71 ***	15.27 ± 0.08	0.41 ± 0.11 *
Lasang	20.54 ± 9.81	5.14 ± 2.45 *	15.20 ± 0.21	0.77 ± 0.38
Legato	28.26 ± 8.12	7.07 ± 2.03	16.62 ± 0.26 ***	1.19 ± 0.35
Linus	45.60 ± 2.26	11.40 ± 0.57	11.26 ± 0.32 ***	1.31 ± 0.03
Magura	39.60 ± 8.49	9.90 ± 2.12	16.98 ± 0.15 ***	1.73 ± 0.35
Monsun	38.20 ± 9.33	9.55 ± 2.33	14.41 ± 0.36 ***	1.43 ± 0.30
Nodula	31.20 ± 9.05	7.80 ± 2.26	15.84 ± 0.63	1.22 ± 0.41
Poljanka	35.00 ± 1.41	8.75 ± 0.35	16.81 ± 0.42 ***	1.47 ± 0.10
Rezista	36.20 ± 11.03	9.05 ± 2.76	9.42 ± 0.50 ***	0.90 ± 0.30
Sadunai	43.66 ± 16.49	10.92 ± 4.12	13.97 ± 0.31 ***	1.53 ± 0.61
Sigord	52.80 ± 7.35	13.20 ± 1.84	16.42 ± 0.62 **	2.17 ± 0.22
Tedi	26.00 ± 8.49	6.50 ± 2.12	14.28 ± 0.24 ***	0.92 ± 0.29
Titus	43.32 ± 11.71	10.83 ± 2.93	15.95 ± 0.43	1.79 ± 0.42
Tornado	38.30 ± 10.32	9.58 ± 2.58	17.46 ± 0.54 ***	1.68 ± 0.40
Vesna	51.20 ± 12.45	12.80 ± 3.11	17.86 ± 0.16 ***	2.36 ± 0.54
*p*-value	<0.001	<0.001	<0.001	<0.001

Values presented as mean ± SD; */**/***—significance from post hoc tests according to 0.05/0.01/0.001 significance threshold.

**Table 3:** Content of bioactive compounds in diploid cultivars.

Cultivar	Head Weight per Plant HWP (g)	Total Polyphenols TP (mg GAE/100 g)	Total Polyphenols TPP (mg GAE/Plant)	Antioxidant Activity AA (µM Trolox/g)	Antioxidant Activity AAP (µM Trolox/Plant)	Total Flavonoids TF (mg QE/100 g)	Total Flavonoids TFP (mg QE/Plant)
Control ($\overset{-}{X}$)	10.32 ± 2.41	366.29 ± 87.34	37.72 ± 12.46	18.63 ± 10.46	191.29 ± 115.29	10.47 ± 5.66	1.07 ± 0.63
AberChianti	13.61 ± 1.53 ***	336.36 ± 12.02	45.87 ± 6.77 **	7.82 ± 1.55 ***	105.19 ± 9.12 ***	6.93 ± 0.07	0.94 ± 0.10
AberClaret	14.24 ± 1.13 ***	479.64 ± 36.06 ***	68.10 ± 0.28 ***	14.26 ± 2.32	201.70 ± 16.93	31.13 ± 1.77 ***	4.44 ± 0.60 ***
Aita Mare	9.28 ± 0.64	321.14 ± 1.62	29.82 ± 2.20 *	11.62 ± 3.56 ***	106.71 ± 25.66 ***	6.78 ± 0.71	0.63 ± 0.11
Arimaiciai	7.76 ± 0.67 ***	532.50 ± 40.91 ***	41.46 ± 6.73	23.66 ± 2.34 *	184.35 ± 33.94	22.78 ± 0.71 ***	1.77 ± 0.21 ***
Britta	9.65 ± 0.63	319.36 ± 12.02	30.79 ± 0.84 *	13.67 ± 0.55 *	131.79 ± 3.27 *	6.58 ± 0.42 *	0.64 ± 0.08
Callisto (C1)	15.03 ± 0.68 ***	456.57 ± 20.61 ***	68.67 ± 6.18 ***	11.26 ± 3.88 ***	170.43 ± 65.96	8.73 ± 1.63	1.32 ± 0.30
Callisto (C2)	12.74 ± 0.24 ***	495.36 ± 13.44 ***	63.11 ± 0.51 ***	15.10 ± 5.05	192.99 ± 68.06	19.18 ± 7.35 ***	2.46 ± 0.98 ***
Corvus	10.49 ± 1.39	466.71 ± 1.82 ***	48.93 ± 6.30 ***	12.60 ± 0.17 **	132.05 ± 15.75 *	20.78 ± 1.70 ***	2.19 ± 0.47 ***
David Liv	11.40 ± 0.56	247.71 ± 27.48 ***	28.15 ± 1.75 ***	34.20 ± 2.51 ***	389.05 ± 9.46 ***	17.38 ± 1.98 ***	1.99 ± 0.32 ***
Diadem	13.22 ± 1.50 ***	358.86 ± 3.23	47.46 ± 5.81 ***	10.80 ± 2.07 ***	144.36 ± 43.63	8.33 ± 0.35	1.10 ± 0.08
Dimanche	17.70 ± 1.82 ***	367.14 ± 34.35	64.66 ± 0.61 ***	8.98 ± 3.04 ***	156.08 ± 37.38	12.28 ± 1.84	2.19 ± 0.55 ***
Diplo	13.28 ± 0.29 ***	537.43 ± 0.01 ***	71.39 ± 1.57 ***	14.82 ± 6.77	197.80 ± 94.25	12.28 ± 4.10	1.64 ± 0.58 **
Diplomat	12.50 ± 0.65 ***	410.43 ± 3.43	51.32 ± 3.09 ***	14.06 ± 3.58	176.89 ± 53.90	10.93 ± 3.18	1.38 ± 0.47
Dipper	9.16 ± 0.48	270.00 ± 3.03 ***	24.74 ± 1.58 ***	9.86 ± 0.70 ***	90.11 ± 1.65 ***	6.68 ± 0.01	0.61 ± 0.03
Discovery	14.61 ± 1.51 ***	375.21 ± 12.02	54.90 ± 7.42 ***	10.28 ± 1.68 ***	148.94 ± 9.00	6.58 ± 0.85 *	0.97 ± 0.22
Dizstende	6.95 ± 1.20 ***	599.86 ± 23.03 ***	41.53 ± 5.57	22.50 ± 1.64	155.28 ± 15.50	15.68 ± 2.55 **	1.07 ± 0.01
Essi	10.46 ± 2.05	177.43 ± 115.16 ***	17.37 ± 8.40 ***	12.43 ± 1.66 **	128.27 ± 8.18 **	7.83 ± 1.48	0.80 ± 0.01
Flora	11.09 ± 0.90	240.00 ± 16.97 ***	26.54 ± 0.29 ***	30.28 ± 2.92 ***	334.47 ± 5.06 ***	10.98 ± 5.09	1.24 ± 0.66
Gandalf	7.82 ± 0.23 ***	455.36 ± 12.63 ***	35.60 ± 2.05	27.63 ± 2.34 ***	215.65 ± 11.83	6.68 ± 1.70	0.52 ± 0.12 *
GKT Junior	11.17 ± 0.72	396.00 ± 7.27	44.27 ± 3.68	34.71 ± 2.66 ***	386.82 ± 4.55 ***	7.93 ± 1.34	0.88 ± 0.09
Gloria Mestnaia	10.08 ± 0.85	312.00 ± 8.08 *	31.41 ± 1.83	32.78 ± 1.73 ***	329.63 ± 10.29 ***	6.58 ± 0.57 *	0.67 ± 0.11
Granta	9.71 ± 0.21	363.00 ± 108.89	35.11 ± 9.80	35.24 ± 1.83 ***	341.85 ± 10.26 ***	11.53 ± 0.92	1.12 ± 0.06
Justin	8.82 ± 0.16	408.00 ± 0.01	35.98 ± 0.65	7.02 ± 2.34 ***	62.05 ± 21.73 ***	6.78 ± 1.27	0.60 ± 0.12
Kindia	11.85 ± 0.88	443.57 ± 21.82 ***	52.66 ± 6.48 ***	10.52 ± 1.00 ***	125.13 ± 21.07 **	11.98 ± 0.42	1.42 ± 0.05
Kornicevskij	9.96 ± 0.64	284.14 ± 7.88 ***	28.27 ± 1.04 ***	13.19 ± 2.09 *	130.67 ± 12.37 **	9.43 ± 0.21	0.94 ± 0.04
Kuhn	11.27 ± 1.31	306.00 ± 16.97 *	34.60 ± 5.91	7.68 ± 3.32 ***	84.43 ± 27.38 ***	6.53 ± 0.07 *	0.74 ± 0.08
L.E. 116	8.43 ± 0.43 **	336.86 ± 7.27	28.42 ± 2.08 **	15.31 ± 1.17	129.35 ± 16.52 **	8.23 ± 0.49	0.69 ± 0.08
Leliceni	11.79 ± 0.25	342.00 ± 25.46	40.29 ± 2.14	9.38 ± 3.22 ***	110.13 ± 35.64 ***	5.78 ± 0.57 **	0.68 ± 0.05
Lemmon	8.07 ± 0.35 ***	275.64 ± 12.02 ***	22.27 ± 1.94 ***	5.20 ± 1.66 ***	41.69 ± 11.56 ***	7.33 ± 1.06	0.59 ± 0.11
Livada Ralu	10.03 ± 0.09	400.00 ± 14.14	40.14 ± 1.76	35.28 ± 1.77 ***	353.90 ± 14.73 ***	19.48 ± 0.57 ***	1.95 ± 0.07 ***
Livada Sara	12.92 ± 0.19 ***	482.14 ± 60.00 ***	62.36 ± 8.66 ***	28.04 ± 2.88 ***	362.10 ± 32.00 ***	12.83 ± 6.01	1.65 ± 0.75 **
Lucrum	11.68 ± 1.44	345.00 ± 21.21	40.15 ± 2.50	31.00 ± 2.47 ***	360.44 ± 15.91 ***	10.93 ± 2.90	1.26 ± 0.18
Manuela	6.03 ± 0.05 ***	306.43 ± 37.78 *	18.48 ± 2.13 ***	24.56 ± 2.75 **	148.13 ± 15.45	5.73 ± 0.35 **	0.35 ± 0.02 ***
Marcom	10.19 ± 1.02	257.14 ± 21.01 ***	26.09 ± 0.49 ***	12.78 ± 1.62 **	129.32 ± 3.45 **	7.58 ± 0.28	0.77 ± 0.05
Marga Liv	9.99 ± 0.78	420.43 ± 0.01 *	42.00 ± 3.26	34.79 ± 2.36 ***	346.66 ± 3.44 ***	6.13 ± 0.35 *	0.61 ± 0.01
Marieta	5.24 ± 0.04 ***	390.86 ± 7.27	20.47 ± 0.24 ***	10.51 ± 2.98 ***	55.00 ± 15.24 ***	12.83 ± 0.35	0.67 ± 0.02
Marino	9.38 ± 0.18	340.00 ± 4.04	31.89 ± 0.99	7.26 ± 1.62 ***	68.19 ± 16.52 ***	7.03 ± 1.77	0.66 ± 0.18
Mercury	6.31 ± 0.41 ***	270.00 ± 29.09 ***	16.99 ± 0.74 ***	6.66 ± 1.23 ***	41.77 ± 5.04 ***	7.58 ± 0.42	0.48 ± 0.01 **
Merian	6.85 ± 0.71 ***	334.29 ± 0.01	22.89 ± 2.37 ***	31.31 ± 1.66 ***	213.81 ± 10.88	7.08 ± 0.57	0.48 ± 0.01 **
Merviot (C1)	5.91 ± 0.16 ***	340.71 ± 34.55	20.09 ± 1.49 ***	8.04 ± 1.83 ***	47.64 ± 12.10 ***	7.73 ± 0.35	0.46 ± 0.01 **
Merviot (C2)	7.12 ± 0.13 ***	348.43 ± 27.27	24.83 ± 2.39 ***	31.70 ± 2.06 ***	225.87 ± 18.71	9.23 ± 2.47	0.66 ± 0.19
Mestecăniș	10.11 ± 1.64	268.29 ± 1.21 ***	27.11 ± 4.28 ***	16.83 ± 0.53	170.53 ± 32.95	9.08 ± 1.13	0.91 ± 0.03
Metis	10.96 ± 0.14	375.71 ± 2.02	41.17 ± 0.31	10.03 ± 2.19 ***	110.06 ± 25.40 ***	8.13 ± 0.64	0.89 ± 0.06
Mistral	10.78 ± 0.24	317.71 ± 16.16	34.24 ± 0.99	34.34 ± 3.11 ***	370.63 ± 41.65 ***	9.48 ± 0.99	1.02 ± 0.13
Montcalme	8.64 ± 0.37 *	412.29 ± 68.29	35.50 ± 4.38	7.96 ± 1.87 ***	68.45 ± 13.19 ***	14.53 ± 2.47 *	1.25 ± 0.16
Nemaro	7.16 ± 0.04 ***	312.00 ± 4.24 *	22.33 ± 0.18 ***	11.51 ± 1.49 ***	82.35 ± 10.21 ***	17.38 ± 0.28 ***	1.24 ± 0.01
Niderheicher	10.73 ± 0.61	335.14 ± 6.87	35.98 ± 2.79	30.39 ± 2.36 ***	325.34 ± 6.66 ***	7.48 ± 0.99	0.80 ± 0.06
Noe	11.07 ± 0.17	348.86 ± 4.44	38.63 ± 1.10	10.40 ± 0.79 ***	115.23 ± 10.57 ***	7.53 ± 0.07	0.83 ± 0.02
Oden Walder	9.07 ± 0.33	331.71 ± 10.91	30.06 ± 0.10 *	6.19 ± 2.04 ***	55.79 ± 16.43 ***	6.18 ± 0.57 *	0.56 ± 0.03 *
Otawa	9.66 ± 0.68	368.00 ± 16.16	35.59 ± 4.05	32.99 ± 2.98 ***	317.51 ± 6.46 ***	7.88 ± 0.14	0.76 ± 0.04
Palna	10.95 ± 2.19	347.29 ± 13.74	37.89 ± 6.09	34.50 ± 1.34 ***	376.41 ± 60.76 ***	10.18 ± 1.41	1.10 ± 0.07
Pavo	9.46 ± 0.90	426.21 ± 2.73 *	40.29 ± 3.58	30.84 ± 2.62 ***	290.49 ± 2.99 ***	7.93 ± 0.07	0.75 ± 0.08
Podjavorina	8.93 ± 0.25	449.43 ± 10.51 ***	40.12 ± 0.20	6.78 ± 0.87 ***	60.61 ± 9.46 ***	16.28 ± 0.99 ***	1.45 ± 0.13
Radviliai	6.77 ± 0.30 ***	706.29 ± 6.46 ***	47.83 ± 2.55 ***	28.35 ± 2.26 ***	191.62 ± 6.86	36.23 ± 3.61 ***	2.44 ± 0.14 ***
Raunis	10.99 ± 1.35	400.00 ± 8.08	43.91 ± 4.51	35.16 ± 1.90 ***	385.18 ± 26.50 ***	7.23 ± 1.06	0.79 ± 0.02
Reichersberger	11.59 ± 0.87	301.71 ± 26.67 **	35.09 ± 5.72	15.24 ± 3.98	174.97 ± 32.83	6.58 ± 0.14 *	0.76 ± 0.07
Rotrif	10.87 ± 0.10	350.00 ± 25.25	38.05 ± 3.09	5.90 ± 2.11 ***	63.98 ± 22.38 ***	6.23 ± 0.35 *	0.68 ± 0.04
Sapporo	9.54 ± 0.30	382.29 ± 12.12	36.48 ± 2.30	25.50 ± 1.62 ***	242.95 ± 7.84	13.88 ± 7.50	1.31 ± 0.67
Segur	12.97 ± 0.81 ***	296.57 ± 4.85 **	38.48 ± 3.03	18.91 ± 3.70	243.72 ± 32.60 *	10.33 ± 0.35	1.34 ± 0.13
Sepia	10.06 ± 0.82	351.50 ± 5.76	35.39 ± 3.45	10.11 ± 3.51 ***	100.29 ± 27.04 ***	7.13 ± 0.49	0.72 ± 0.01
Sinope	10.10 ± 0.14	359.79 ± 30.20	36.34 ± 3.54	34.82 ± 2.39 ***	351.66 ± 28.90 ***	9.13 ± 1.63	0.92 ± 0.18
Slatina	10.59 ± 0.03	366.43 ± 1.82	38.79 ± 0.29	12.34 ± 0.74 **	130.60 ± 8.11 **	6.48 ± 0.14 *	0.69 ± 0.02
Suez	9.05 ± 0.17	354.00 ± 33.94	32.05 ± 3.66	9.95 ± 3.02 ***	90.26 ± 28.96 ***	16.48 ± 0.57 ***	1.49 ± 0.02
Tabor	7.86 ± 0.91 ***	274.29 ± 6.06 ***	21.52 ± 2.02 ***	11.02 ± 3.36 ***	85.02 ± 16.34 ***	10.53 ± 0.64	0.82 ± 0.05
Tășnad	11.49 ± 0.37	342.86 ± 12.12	39.38 ± 0.14	30.94 ± 3.32 ***	354.88 ± 26.80 ***	9.88 ± 1.84	1.14 ± 0.25
Tinu Liv	11.28 ± 0.08	405.00 ± 4.24	45.69 ± 0.81 *	26.95 ± 3.26 ***	304.18 ± 39.01 ***	5.63 ± 0.07 **	0.63 ± 0.01
Verdi	15.08 ± 0.34 ***	235.86 ± 10.51 ***	35.56 ± 0.78	34.64 ± 2.45 ***	522.99 ± 48.86 ***	7.43 ± 0.49	1.12 ± 0.05
Violeta	9.70 ± 0.66	273.86 ± 27.27 ***	26.49 ± 0.83 ***	8.76 ± 1.19 ***	84.64 ± 5.71 ***	8.03 ± 0.35	0.78 ± 0.09
Violetta R.v.P.	14.22 ± 0.91 ***	353.57 ± 12.73	50.23 ± 1.39 ***	9.80 ± 2.62 ***	138.24 ± 28.39 *	8.28 ± 0.42	1.18 ± 0.14
Vyciai	9.02 ± 0.84	444.00 ± 25.46 ***	39.92 ± 1.42	13.95 ± 2.15	124.87 ± 7.70 **	6.83 ± 1.06	0.61 ± 0.04
*p*-value	<0.001	<0.001	<0.001	<0.001	<0.001	<0.001	<0.001

C1 & C2 (Callisto and Merviot) are the same cultivar received from different centers. Values presented as mean ± SD; */**/***—significance from post hoc tests according to 0.05/0.01/0.001 significance threshold.

**Table 4:** Content of bioactive compounds in tetraploid cultivars.

Cultivar	Head Weight per Plant HWP (g)	Total Polyphenols TP (mg GAE/100 g)	Total Polyphenols TPP (mg GAE/Plant)	Antioxidant Activity AA (µM Trolox/g)	Antioxidant Activity AAP (µM Trolox/Plant)	Total Flavonoids TF (mg QE/100 g)	Total Flavonoids TFP (mg QE/Plant)
Control ($\overset{-}{X}$)	10.57 ± 4.27	413.62 ± 68.39	43.51 ± 18.92	17.41 ± 9.88	167.43 ± 96.27	10.28 ± 5.41	1.00 ± 0.42
Amos	9.40 ± 0.71 *	434.00 ± 5.66	40.80 ± 3.61	19.04 ± 2.11	179.68 ± 33.36	10.48 ± 1.13	0.98 ± 0.03
Beskyd	10.78 ± 0.18	395.14 ± 10.91	42.60 ± 0.48	21.62 ± 0.66	233.06 ± 3.31 *	12.88 ± 3.39	1.39 ± 0.34 *
Bivoj	18.12 ± 1.03 ***	506.00 ± 4.44 **	91.66 ± 4.43 ***	12.88 ± 4.60	235.79 ± 96.68 *	8.08 ± 2.26	1.48 ± 0.49 **
Dolina	12.63 ± 0.88 ***	380.07 ± 5.15	48.01 ± 3.99	34.86 ± 1.70 ***	439.34 ± 9.18 ***	10.63 ± 1.48	1.34 ± 0.09
Ilte	8.84 ± 0.40 ***	564.29 ± 22.22 ***	49.91 ± 4.23	17.62 ± 1.98	156.06 ± 24.56	11.38 ± 0.42	1.01 ± 0.08
Lars	7.51 ± 0.50 ***	364.57 ± 11.31	27.36 ± 0.96 **	18.16 ± 0.94	136.67 ± 16.08	11.18 ± 0.99	0.84 ± 0.13
Lasang	6.81 ± 0.65 ***	380.57 ± 4.85	25.91 ± 2.16 ***	35.34 ± 1.62 ***	240.22 ± 12.08 **	12.08 ± 1.98	0.82 ± 0.06
Legato	4.68 ± 0.01 ***	474.43 ± 52.73	22.22 ± 2.44 ***	16.20 ± 2.70	75.89 ± 12.53 ***	30.88 ± 4.24 ***	1.45 ± 0.20 **
Linus	5.59 ± 0.69 ***	409.14 ± 0.01	22.87 ± 2.83 ***	35.36 ± 1.66 ***	197.08 ± 15.20	9.58 ± 1.27	0.54 ± 0.14 **
Magura	10.70 ± 0.04	318.93 ± 5.76 **	34.12 ± 0.74	31.87 ± 2.38 ***	340.94 ± 26.69 ***	6.53 ± 0.35 *	0.70 ± 0.04
Monsun	10.86 ± 0.42	361.29 ± 9.09	39.25 ± 2.51	10.84 ± 2.28 **	117.25 ± 20.20	7.28 ± 0.85	0.79 ± 0.06
Nodula	8.48 ± 0.41 ***	381.00 ± 33.94	32.24 ± 1.31 *	15.52 ± 2.41	131.15 ± 14.08	7.38 ± 0.28	0.63 ± 0.05 *
Poljanka	7.10 ± 0.20 ***	534.86 ± 29.09 ***	37.92 ± 1.00	17.83 ± 4.02	126.11 ± 24.97	7.83 ± 1.63	0.55 ± 0.10 **
Rezista	5.60 ± 0.59 ***	351.00 ± 27.27	19.58 ± 0.55 ***	16.20 ± 5.98	89.00 ± 23.91 **	10.78 ± 1.56	0.60 ± 0.02 *
Sadunai	14.64 ± 0.85 ***	480.57 ± 5.86 *	70.39 ± 4.95 ***	7.60 ± 1.47 ***	110.69 ± 15.07	14.18 ± 4.24 *	2.06 ± 0.50 ***
Sigord	10.36 ± 0.59	343.57 ± 13.13 *	35.62 ± 3.37	5.54 ± 1.30 ***	57.72 ± 16.72 ***	5.63 ± 0.64 **	0.58 ± 0.10 **
Tedi	8.11 ± 0.38 ***	455.14 ± 27.27	36.97 ± 3.95	7.14 ± 2.09 ***	57.49 ± 14.26 ***	7.43 ± 0.21	0.60 ± 0.05 *
Titus	14.92 ± 0.51 ***	413.57 ± 27.27	61.77 ± 6.17 ***	7.14 ± 3.34 ***	105.61 ± 46.16 *	7.43 ± 0.07	1.11 ± 0.03
Tornado	18.75 ± 1.06 ***	342.86 ± 12.12 *	64.35 ± 5.91 ***	11.06 ± 3.07 **	205.67 ± 45.90	8.28 ± 1.13	1.55 ± 0.12 ***
Vesna	17.46 ± 0.05 ***	381.43 ± 139.40	66.57 ± 24.14 ***	6.48 ± 2.72 ***	113.13 ± 47.07	5.83 ± 0.78 **	1.02 ± 0.13
*p*-value	<0.001	<0.001	<0.001	<0.001	<0.001	<0.001	<0.001

Values presented as mean ± SD; */**/***—significance from post hoc tests according to 0.05/0.01/0.001 significance threshold.

**Table 5:** Breeding-oriented cultivar recommendations based on an integrated evaluation of forage and medicinal traits.

Breeding Objective	Ploidy	Recommended Cultivars	Main Justification
Forage-oriented breeding	2n	Diadem; Otawa; Discover; AberChianti; Tășnad.	High dry matter yield (DMY), green matter yield (GMY), crude protein (CP), and protein yield (PY). Diploids provide stability and forage quality, while tetraploids ensure superior biomass accumulation and higher total yield potential.
	4n	Vesna; Bivoj; Sigord; Dolina; Beskyd.	
Medicinal-oriented breeding	2n	Radviliai; Dizstende; Diplo; Livada Sara; Verdi.	Superior concentrations and/or per-plant productivity of polyphenols (TP, TPP), flavonoids (TF, TFP), and antioxidant activity (AA, AAP). Diploids dominate in phytochemical concentration, while tetraploids strongly enhance the per-plant bioactive yield.
	4n	Ilte; Poljanka; Sadunai; Tornado; Linus.	
Dual-purpose breeding	2n	AberClaret; Callisto (C1); Diplo; David Liv; Verdi.	Balanced performance across agronomic traits (DMY, PY) and medicinal traits (TP, TF, AA). These cultivars combine acceptable forage productivity with strong or moderate bioactive compound production, making them suitable for multifunctional breeding programs.
	4n	Bivoj; Vesna; Sigord; Dolina; Poljanka.	

Among the identified groups, dual-purpose candidates are of particular breeding interest because they combine acceptable forage productivity with favorable phytochemical characteristics. These cultivars may represent valuable parental material for breeding programs aiming to integrate forage value and functional bioactive potential within the same cultivar.

## Data Availability

The original contributions presented in this study are included in the article/Supplementary Material. Further inquiries can be directed to the corresponding author.

## Supplementary Materials

The following supporting information can be downloaded at: https://www.mdpi.com/article/10.3390/plants15121910/s1, Table S1: Diploid and tetraploid cultivar list.

## Abbreviations

PCA	Principal component analysis
CP	Crude protein
DM	Dry matter
NDF	Neutral detergent fiber
ADF	Acid detergent fiber
GMY	Green matter yield
DMY	Dry matter yield
PY	Protein yield
HWP	Head weight per plant
TP	Total polyphenols
AA	Antioxidant activity
TF	Total flavonoids
SD	Standard deviation
RCBD	Randomized complete block design
UASVM	University of Agricultural Sciences and Veterinary Medicine
KMO	Kaiser–Meyer–Olkin
