# Supplementary material for: Integrated Evaluation of Agronomic and Phytochemical Traits in Red Clover (Trifolium pratense L.) for Dual-Purpose Breeding
Source: Plants (Basel). 2026 Jun 20;15(12):1910. doi: 10.3390/plants15121910 (PMC13306861; doi:10.3390/plants15121910)
Supplement: Supplementary file 1 [file plants-15-01910-s001.zip › plants-4355288-supplementary.pdf]

Supplementary Table S1 – diploid and tetraploid cultivar list

Nr.crt.	Cultivar	Ploidy level	Geographical region	Seeds' country of origin	Breeding institution or germplasm source
1	AberChianti	2n	Western Europe	United Kingdom (UK)	Aberystwyth University
2	AberClaret	2n	Western Europe	United Kingdom (UK)	Aberystwyth University
3	Aita Mare	2n	Southern Europe	Romania	SCDA Livada germplasm collection
4	Amos	4n	Central Europe	Czech Republic	DLF Seeds
5	Arimaiciai	2n	Northern Europe	Lithuania	Institute of Agriculture Lithuanian Research Centre for Agriculture and Forestry
6	Beskyd	4n	Central Europe	Czech Republic	DLF Seeds
7	Bivoj	4n	Central Europe	Czech Republic	DLF Seeds
8	Britta	2n	Northern Europe	Sweden	SCDA Livada germplasm collection
9	Callisto (Centre 1)	2n	Northern Europe	Denmark	DLF Seeds
10	Callisto (Centre 2)	2n	Central Europe	Czech Republic	DLF Seeds
11	Corvus	2n	Central Europe	Switzerland	Agroscope
12	David Liv	2n	Southern Europe	Romania	SCDA Livada
13	Diadem	2n	Western Europe	France	Agri Obtention
14	Dimanche	2n	Western Europe	France	Agri Obtention
15	Diplo	2n	Western Europe	France	Agri Obtention
16	Diplomat	2n	Central Europe	Germany	Hans Georg Lembke KG/NPZ
17	Dipper	2n	Western Europe	France	SCDA Livada germplasm collection
18	Discovery	2n	Western Europe	France	Agri Obtention
19	Dizstende	2n	Northern Europe	Latvia	Institute of Agricultural Resources and Economics
20	Dolina	4n	Central Europe	Czech Republic	DLF Seeds
21	Essi	2n	Northern Europe	Sweden	SCDA Livada germplasm collection
22	Flora	2n	Southern Europe	Romania	SCDA Livada germplasm collection
23	Gandalf	2n	Northern Europe	Norway	Graminor AS
24	GKT Junior	2n	Central Europe	Hungary	SCDA Livada germplasm collection
25	Gloria Mestnaia	2n	Eastern Europe	Ukraine	SCDA Livada germplasm collection
26	Granta	2n	Western Europe	United Kingdom (UK)	SCDA Livada germplasm collection
27	Ilte	4n	Northern Europe	Estonia	Estonian Crop Research Institute
28	Justin	2n	Western Europe	France	SCDA Livada germplasm collection
29	Kindia	2n	Western Europe	France	Agri Obtention
30	Kornicevskij	2n	Eastern Europe	Russia	SCDA Livada germplasm collection
31	Kuhn	2n	Western Europe	Netherlands	SCDA Livada germplasm collection
32	L.E. 116	2n	South America	Uruguay	INIA-La Estanzuela
33	Lars	4n	Northern Europe	Norway	Graminor AS
34	Lasang	4n	Northern Europe	Norway	Graminor AS

Supplementary Table S1 – diploid and tetraploid cultivar list

Nr.crt.	Cultivar	Ploidy level	Geographical region	Seeds' country of origin	Breeding institution or germplasm source
35	Legato	4n	Northern Europe	Norway	Graminor AS
36	Leliceni	2n	Southern Europe	Romania	SCDA Livada germplasm collection
37	Lemmon	2n	Western Europe	Belgium	ILVO Plant - Toegepaste Genetica en Veredeling
38	Linus	4n	Northern Europe	Norway	Graminor AS
39	Livada Ralu	2n	Southern Europe	Romania	SCDA Livada
40	Livada Sara	2n	Southern Europe	Romania	SCDA Livada
41	Lucrum	2n	Western Europe	France	SCDA Livada germplasm collection
42	Magura	4n	Central Europe	Slovakia	Centrum výskumu rastlinnej výroby Pieš tany
43	Manuela	2n	Central Europe	Slovakia	Centrum výskumu rastlinnej výroby Pieš tany
44	Marcom	2n	Western Europe	France	SCDA Livada germplasm collection
45	Marga Liv	2n	Southern Europe	Romania	SCDA Livada
46	Marieta	2n	Central Europe	Slovakia	Centrum výskumu rastlinnej výroby Pieš tany
47	Marino	2n	Western Europe	France	SCDA Livada germplasm collection
48	Mercury	2n	Western Europe	Belgium	Rijksstation voor Plantenveredeling
49	Merian	2n	Western Europe	Belgium	ILVO Plant - Toegepaste Genetica en Veredeling
50	Merviot (Centre 1)	2n	Western Europe	Belgium	ILVO Plant - Toegepaste Genetica en Veredeling
51	Merviot (Centre 2)	2n	Western Europe	Belgium	Rijksstation voor Plantenveredeling
52	Mestecăniş	2n	Southern Europe	Romania	SCDA Livada germplasm collection
53	Metis	2n	Northern Europe	Denmark	DLF Seeds
54	Mistral	2n	Western Europe	France	SCDA Livada germplasm collection
55	Monsun	4n	Central Europe	Germany	Saatzucht Steinach GmbH & Co KG
56	Montcalme	2n	Western Europe	France	Agroscope
57	Nemaro	2n	Central Europe	Germany	Saatzucht Steinach GmbH & Co KG
58	Niderheicher	2n	Central Europe	Germany	SCDA Livada germplasm collection
59	Nodula	4n	Central Europe	Czech Republic	DLF Seeds
60	Noe	2n	Western Europe	France	SCDA Livada germplasm collection
61	Oden Walder	2n	Central Europe	Germany	SCDA Livada germplasm collection
62	Otawa	2n	North America	Canada	SCDA Livada germplasm collection
63	Palna	2n	Western Europe	France	SCDA Livada germplasm collection
64	Pavo	2n	Central Europe	Switzerland	Agroscope
65	Podjavorina	2n	Central Europe	Slovakia	Centrum výskumu rastlinnej výroby Pieš tany
66	Poljanka	4n	Southern Europe	Slovenia	Kmetijski inš titut Slovenije
67	Radviliai	2n	Northern Europe	Lithuania	Institute of Agriculture Lithuanian Research Centre for Agriculture and Forestry
68	Raunis	2n	Northern Europe	Latvia	Institute of Agricultural Resources and Economics

Supplementary Table S1 – diploid and tetraploid cultivar list

Nr.crt.	Cultivar	Ploidy level	Geographical region	Seeds' country of origin	Breeding institution or germplasm source
69	Reichersberger	2n	Central Europe	Austria	SCDA Livada germplasm collection
70	Rezista	4n	Central Europe	Czech Republic	DLF Seeds
71	Rotrif	2n	Southern Europe	Romania	SCDA Livada
72	Sadunai	4n	Northern Europe	Lithuania	Institute of Agriculture Lithuanian Research Centre for Agriculture and Forestry
73	Sapporo	2n	Western Europe	France	SCDA Livada germplasm collection
74	Segur	2n	Western Europe	France	SCDA Livada germplasm collection
75	Sepia	2n	North America	USA - United States	SCDA Livada germplasm collection
76	Sigord	4n	Central Europe	Slovakia	Centrum výskumu rastlinnej výroby Pieš tany
77	Sinope	2n	Northern Europe	Denmark	DLF Seeds
78	Slatina	2n	Central Europe	Slovakia	Centrum výskumu rastlinnej výroby Pieš tany
79	Suez	2n	Northern Europe	Denmark	DLF Seeds
80	Tabor	2n	Central Europe	Czech Republic	SCDA Livada germplasm collection
81	Tășnad	2n	Southern Europe	Romania	SCDA Livada germplasm collection
82	Tedi	4n	Western Europe	France	INRA (Institut National de la Recherche Agronomique), C/O Agri Obtentions S.A.
83	Tinu Liv	2n	Southern Europe	Romania	SCDA Livada
84	Titus	4n	Central Europe	Germany	Saatzucht Steinach GmbH & Co KG
85	Tornado	4n	Central Europe	Germany	Saatzucht Steinach GmbH & Co KG
86	Verdi	2n	Western Europe	France	SCDA Livada germplasm collection
87	Vesna	4n	Central Europe	Czech Republic	DLF Seeds
88	Violeta	2n	Western Europe	France	SCDA Livada germplasm collection
89	Violetta R.v.P.	2n	Western Europe	Belgium	ILVO Plant - Toegepaste Genetica en Veredeling
90	Vyciai	2n	Northern Europe	Lithuania	Institute of Agriculture Lithuanian Research Centre for Agriculture and Forestry
